# The Effect of Daily Intake of Selenium, Vitamin E and Folic Acid on Sperm Parameters in Males with Idiopathic Infertility: A Single-Blind Randomized Controlled Clinical Trial

**DOI:** 10.22074/IJFS.2021.6236

**Published:** 2021-01-27

**Authors:** Rezvan Bahmyari, Ali Ariafar, Mehrab Sayadi, Shirzad Hossieni, Sara Azima

**Affiliations:** 1 Department of Midwifery, School of Nursing and Midwifery, Shiraz University of Medical Sciences, Shiraz, Iran; 2 Department of Urology, School of Medicine, Shiraz University of Medical Sciences, Shiraz, Iran; 3 Cardiovascular Research Center, Shiraz University of Medical Sciences, Shiraz, Iran; 4 Department of Anatomy, School of Medicine, Tehran University of Medical Sciences, Tehran, Iran; 5 Embryology Department, Shiraz Fertility Center, Shiraz, Iran

**Keywords:** Folic Acid, Parameters, Selenium, Spermatozoa, Vitamin E

## Abstract

**Background:**

Male infertility may originate from a wide spectrum of conditions while in 30-40 percent of cases, no signifi- cant reason can be identified. Thereby, it is recognized as male idiopathic infertility. This study was undertaken to investigate the effect of daily intake of selenium, vitamin E and folic acid on sperm parameters in males with idiopathic infertility.

**Materials and Methods:**

Seventy infertile men were selected to participate in this single-blind, randomized con- trolled clinical trial using convenience sampling. They were equally divided into two groups via permuted block randomization method. The intervention group received selenium tablet (200 µg per day, oral), vitamin E capsule (400 IU per day, oral) and folic acid tablet (5 mg per day, oral).The placebo group received matching placebo for three months. Semen volume, total sperm motility, sperm concentration, progressive sperm motility, normal sperm morphology, sperm motility index (SMI) and functional sperm concentration (FSC) were assessed by sperm quality analyzer-v (SQAV) before and after the intervention. Paired t test, and independent t test were used to compare the results within and between the groups, respectively. The IBM SPSS V.16.005 was used for data analysis. A P<0.05 was considered statistically significant.

**Results:**

After three months, according to within-group analysis, a significant difference was found in mean SMI (P=0.007) and FSC (P=0.001) in the intervention group. According to between-group analysis, no significant difference was found in mean semen volume (P=0.610), sperm concentration (P=0.126), total sperm motility (P=0.765), progres- sive sperm motility (P=0.767), normal sperm morph (P=0.403), SMI (P=0.556) or FSC (P=0.706) between the groups.

**Conclusion:**

Consumption of selenium, vitamin E and folic acid in infertile men with asthenozoospermia was not effective (Registration number: IRCT2017012432153N1).

## Introduction

Infertility is characterized as “not being able to get pregnant, after at least one year of regular unprotected sex” (1). In fact, 15% of couples worldwide suffer from infertility (2). Male factor contributes to 20 to 70% of infertility cases and the percentage of infertility solely due to male factor is estimated to be 2.5 to 12% (3). Male infertility may be originated from a wide spectrum of conditions such as anatomy and genetic disorders, neurological or systemic diseases, trauma, infection, iatrogenic injury, gonadotoxins and formation of sperm antibodies (4). In nearly 30 to 40% of infertility cases, there is an abnormality in semen parameters in terms of motility, morphology or concentration, in at least two semen analyses while no significant reason can be detected. Therefore, this type of infertility is identified as male idiopathic infertility (4, 5). Accumulating research has clarified the fundamental role of low levels of reactive oxygen species (ROS) in intracellular signaling which is responsible for spermatozoa maturation, capacitation, hyperactivation, acrosomal reaction and oocyte fusion (6). Several studies have reported that elevated seminal ROS levels exist in 30-80% of infertile men (7). In fact, spermatozoa membrane is rich in poly unsaturated fatty acid; hence, it is vulnerable to the detrimental effects of excessive amounts of ROS which lead to lipid peroxidation, loss of membrane integrity, increased membrane permeability, reduction of sperm motility, structural DNA damage and apoptosis (8). Experimental investigation on sub-fertile men have indicated lower levels of antioxidants in the semen as compared with fertile men (9). According to recent research, to confront excessive generation of ROS, various intrinsic antioxidants and extrinsic antioxidants have been applied which controlled the detrimental production of ROS and prevented their side effects (10). Proper spermatogenesis requires two selenoproteins: phospholipid peroxide glutathione peroxidase (PHGPX) and selenoprotein P. In the testis, selenium works in the form of PHGPX known as a selenium-dependent antioxidant enzyme. The most considerable role of this agent is protecting plasmatic membrane of mature spermatozoa against the attack of free radicals. This protein also organizes 50% of the material of mitochondrial mid-piece of spermatozoa; thus, in cases of selenium deficiency, reduced motility of spermatozoa due to abnormality in the morphology of spermatozoon midpiece are detected (11). Vitamin E is a fat-soluble vitamin that restrains free radicals which induce damage to cell membranes, prevents lipid peroxidation and improves the activity of other antioxidants, thereby decreasing seminal ROS in infertile males. Also, there are some epidemiological data that support a direct relation between improvement of seminal parameters and increased dietary intake of vitamin E (12). Folic acid, as a synthetic form of folate, efficiently scavenges free radicals and has been introduced as an effective factor for reduction of ROS in seminal fluid (13). Therefore, the present study aimed to investigate the effects of daily intake of selenium, vitamin E and folic acid as probably the most effective antioxidant components on sperm parameters in males with idiopathic infertility.

## Materials and Methods

This single-blind randomized controlled clinical trial was carried out on 70 men who met the inclusion criteria, and were diagnosed as idiopathic infertile patients attending the clinics of urology (affiliated to Shiraz University of Medical Sciences, Shiraz, Iran) from June 2016 to September 2018. The approval ID for this interventional study was obtained from the Ethics Committee of Shiraz University of Medical Sciences (IR.SUMS.REC.1395.160) and the study was registered with clinical trial registry number IRCT2017012432153N1. Written informed consent was taken from all the study participants.

According to the WHO guideline (2010), patients were respectively diagnosed as oligozoospermia, asthenozoospermia, teratozoospermia, if:

- Sperm concentration (sperm numbers per one milliliter of semen) lower than 15 M/ml and higher than 5 M/ml.

- Total sperm motility lower than 40% or progressive sperm motility lower than 32%.

- Percentage of sperm with normal morphology <4%.

Patients with oligo, astheno, terato or oligoasthenoteratospermia [Based on the WHO guideline (2010)] who attended the clinics of urology were recruited if they met the following inclusion criteria: willingness to participate in the study; not being able to get pregnant after at least one year of regular unprotected sex; abnormal seminal analysis results [confirmed after two semen analyses within 3-4 week intervals done after the same sexual abstinence periods (3-5 days)]; absence of underlying causes screened according to pre-testicular, testicular and post-testicular factors (Table 1) (4). We started antioxidant treatment for cases with a history of varicocelectomy at least 3 months later. Also, varicocele recurrence was ruled out again.

**Table 1:** Pre-testicular, testicular and post-testicular factors


Pre-testicular factors	Testicular factors	Post - testicular factors

Kallmann syndrome	Varicocele	Coital
Hyperprolactinaemia	Cryptorchidism	Pharmacological
Pharmacological	Testicular cancer	Retrograde ejaculation
	Radiation	Congenital bilateral absence of the vas deferens
	Chemotherapy	Ejaculatory duct obstruction
	Pharmacological	Seminal vesicle dysfunction
	Genetic azoospermia or Oligospermia	Vasectomy
	Y-chromosome microdeletions	Iatrogenic injury to the vas deferens
	Klinefelter syndrome	Young’s syndrome
	Environmental	Nerve injury
	Anti-sperm antibodies	Spinal cord injury
	Injury or trauma	Retroperitoneal lymph node
	Infection	Systemic disease


The exclusion criteria were: participant’s unwillingness to continue, urogenital infection with antioxidant properties, symptom of an allergy to antioxidant therapy, diagnosis of pre-testicular, testicular or post-testicular factors.

### Study design

Patients who met the inclusion criteria were grouped as either intervention (n=35) or placebo group (n=35), through permuted block randomization method. Patients in the intervention group received selenium tablet (200 µg per day, oral), vitamin E capsule (400 IU per day, oral) and folic acid tablet (5 mg per day, oral).The placebo group received matching placebo (250 mg per day, oral) for three months. The placebo was made in the laboratory of the School of Pharmacy, Shiraz University of Medical Sciences, Shiraz, Iran and sodium starch glycolate 100% was used to make the placebo. In this study, selenium was the product of Webber Naturals Company, Canada, vitamin E was the product of Zahravi pharmaceutical company, Iran, and folic acid was the product of Jalinous pharmaceutical company, Iran. During this period, the patients were trained to limit consumption of red meat and food rich in phytoestrogen such as soybeans (due to their effect on reducing sperm count), and stop smoking (due to the toxic chemicals which can cause mutations) and alcohol consumption (which reduces testosterone level and testicular atrophy). They were also taught not to be exposed to radiation, prolonged heat and the environment of a sauna or Jacuzzi (14). Besides, they were trained to refrain from using a cell phone for more than 11-13 hours per day (15). Also, they were trained to limit consumption of caffeine due to its probable detrimental impact on sperm DNA integrity (16).

After three months, the participants were asked to follow sexual abstinence for 3-5 days, and repeat their semen analysis. Sperm concentration, total sperm motility, progressive sperm motility, sperm morphology, sperm motility index (SMI), and total functional sperm concentration (FSC) were assessed. FSC is the concentration of progressively motile spermatozoa with normal morphology in a semen sample. This is a very difficult parameter to measure manually, since it is required to kill the progressive cells to assess the morphology.

### Semen analysis

For assessment of sperm parameters, the WHO 2010 guidelines were considered and sperm quality analyzer- v (SQAV) instrument and optical microscope (for evaluating morphology) were used. In this study, all the sperm parameters except morphology, were evaluated by SQAV.

By evaluating the accuracy of automated computerized semen analyzer instrument (SQAV and CASA) and conventional manual method according to accuracy and precision, various results were reported.

The advantages of using automated semen analyzer include standardization, speed, precision, automated data recording, fewer human errors and less need for highskilled professionals to perform the analysis. The accuracy of the instrument lies in the fact that it uses a larger sample volume (0.5 ml) in contrast with the volume used in manual analysis and CASA instrument (10-50 µl). The main disadvantage is the inability to carry out a concrete differential morphology assessment. However, this problem has been resolved by integrating manual assessment to detect normal morphology (17).

### Statistical method

Continuous variables with normal distribution, and variables with non-normal distribution are reported as mean ± SD and median (IQR), respectively. The categorical data are presented as numbers (%). For checking the normality of data, Shapiro-Wilks test was used. The difference within and between the two groups was investigated using Paired t test, and independent t test, respectively. Mann-Whitney and Wilcoxon’s tests were used when the assumption of normality was violated. Moreover, we used chi-squared test to compare the distribution of categorical data [The SPSS V. 16.0. with significance level of 0.05 used for data analysis, the product of International Business Machines Corporation (IBM), Chicago, USA]. A P<0.05 was considered significant.

## Results

**Fig.1:**
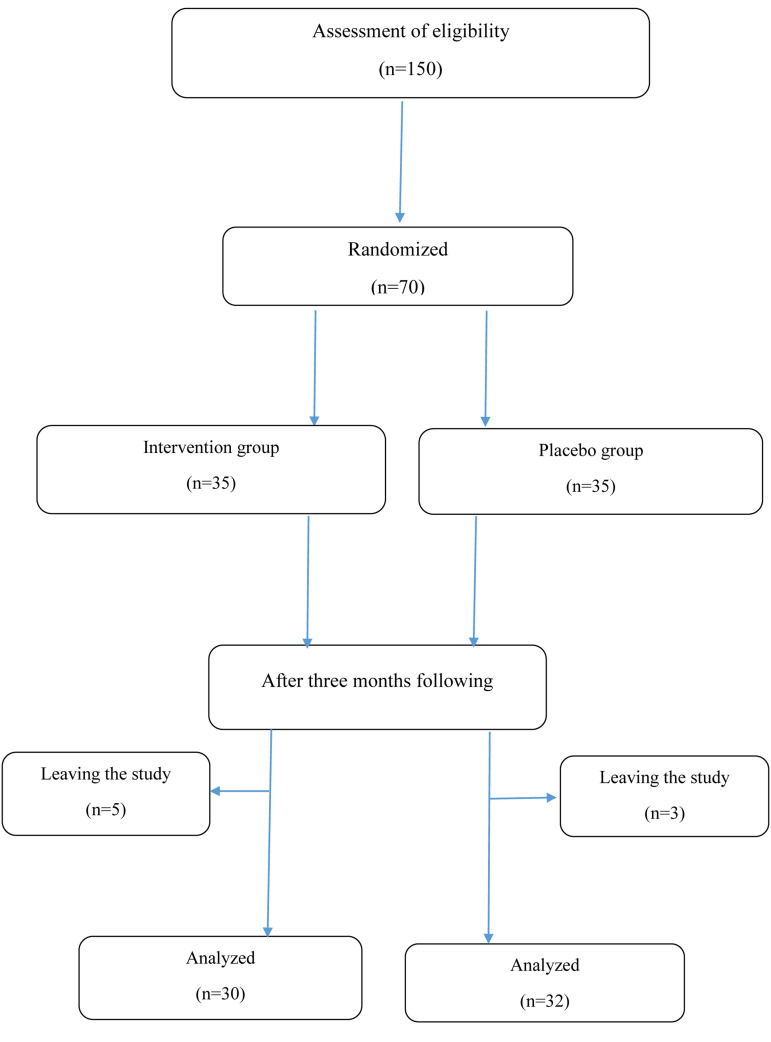
The flowchart of patient selection in this single-blind randomized controlled clinical trial.

A total of 70 patients (aged 18-55 years) with idiopathic infertility, participated in the present study; however, after three months, only 62 patients completed the study: 30 patients in the intervention and 32 in the placebo group (Fig .1).

Table 2 presents demographic and baseline characteristics of the study participants. No significant difference was found in demographic and baseline characteristics among the participants, and the two groups were well balanced: age (P=0.737), duration of marriage (P=0.392), duration of infertility (P=0.070), smoking (P=0.352), alcohol consumption (P=0.591), and level of education (P=0.186). 

Table 3 summarizes the quality of sperm parameters from the baseline to the end of the intervention. No significant difference was found in semen volume (P=0.097), sperm concentration (P=0.270), total sperm motility (P=0.331), progressive sperm motility (P=0.130), normal sperm morphology (P=0.315), SMI (P=0.059) and FSC (P=0.057) between the intervention and control groups at the beginning of the study.

**Fig.2:**
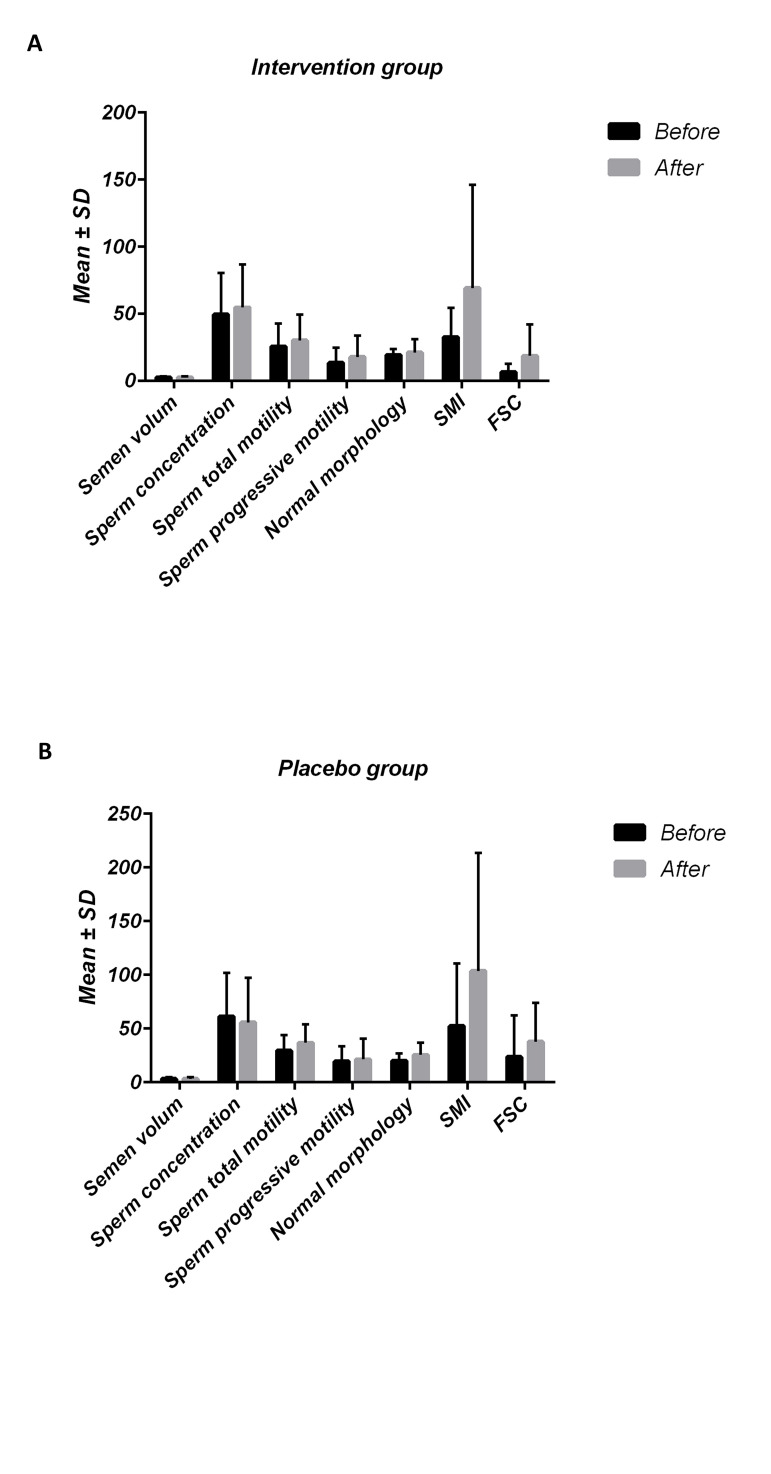
The within and between groups variation of sperm parameters at the end of the study in both groups groups (data expressed as mean ± SD). SMI; Sperm motility index and FSC; Functional Sperm Concentration.

At the end of the intervention (i.e. after three months), within-group analysis indicated significant improvements in SMI (P=0.007) and FSC (P=0.001), but not in other variables in the intervention group. Difference in differences method was used as a statistical technique to investigate the effect of the present intervention by comparing the average change (over time) in the outcome variable in both intervention and placebo groups (i.e.between-group analyses). No significant difference was found in sperm parameters between the intervention and placebo groups at the end of the study (Fig .2, Table 3).

**Table 2:** Demographic and baseline characteristics


Variable		Intervention group n=30	Placebo group n=32	P value^a^

Age (Y)		37.23 ± 7.09	36.65 ± 6.41	0.737
Duration of marriage		9.31 ± 6.23	7.87 ± 5.1	0.392
Duration of infertility		6.03 ± 4.35	4.28 ± 3.05	0.070
Smoking				0.352
	Yes	8 (26.66)	11 (34.38)	
	Never	22 (73.33)	21 (65.62)	
Alcohol consumption				0.591
	Yes	5 (16.66)	5 (15.62)	
	Never	25 (83.33)	27 (84.37)	
Level of education				0.186
	Primary school	1 (3.3)	0 (0)	
	Guidance school	3 (13.3)	2 (6.3)	
	High school	16 (53.3)	12 (37.5)	
	Higher	9 (30)	18 (56.3)	


Data are expressed as mean ± SD or n (%). ^a^ ; Independent t test or chi-squared test.

**Table 3:** The effects of selenium, vitamin E and acid folic on sperm parameters in infertile men


Variable		Intervention group n=30	Placebo group n=32	P value^b^

Volume				
	Baseline	2.50 ± 1.13	3.18 ± 1.62	0.097
	End	2.52 ± 1.06	3.23 ± 1.43	0.038
	Change	0.02 ± 0.84	0.04 ± 0.96	0.610
	P value^a^	0.957	0.430	
Concentration (10^6^/ml)				
	Baseline	49.52 ± 30.98	61.31 ± 40.67	0.270
	End	54.67 ± 32.07	55.79 ± 41.39	0.780
	Change	5.15 ± 28.99	-5.52 ± 23.01	0.126
	P value^a^	0.28	0.170	
Total motility				
	Baseline	25.62 ± 17.11	29.52 ± 14.25	0.331
	End	30.28 ± 19.27	36.68 ± 17.19	0.171
	Change	4.66 ± 17.9	7.15 ± 16.9	0.765
	P value^a^	0.166	0.020	
Progressive motility				
	Baseline	13.5 ± 11.19	19.51 ± 13.92	0.130
	End	17.99 ± 16	21.27 ± 19.19	0.460
	Change	4.1 ± 14.21	1.75 ± 17.61	0.767
	P value^a^	0.120	0.530	
Normal morph				
	Baseline	19.48 ± 4.3	20.02 ± 6.9	0.315
	End	21.18 ± 9.9	25.62 ± 11.33	0.486
	Change	2.69 ± 8.96	5.6 ± 11.7	0.403
P value^a^	0.069	0.339	
SMI				
	Baseline	32.8 ± 21.56	52.33 ± 58.41	0.059
	End	69.2 ± 76.94	103.58 ± 109.79	0.375
	Change	36.4 ± 66.52	51.25 ± 100.38	0.556
	P value^a^	0.007	0.039	
FSC				
	Baseline	6.74 ± 5.98	23.70 ± 38.71	0.057
	End	18.73 ± 23.36	37.74 ± 36.1	0.087
	Change	11.98 ± 20.71	14.04 ± 36.05	0.706
	P value^a^	0.001	0.007	


Data are expressed as mean ± SD. ^a^ ; Paired t test or Wilcoxon test, ^b^; Independent t test or Mann-Whitney U test, SMI; Sperm motility index, and FSC; Functional sperm concentration.

## Discussion

This study aimed to investigate the effect of daily intake of selenium, vitamin E and folic acid on sperm parameters in idiopathic infertile men. At the end of the study, significant changes in total motility were observed in the placebo group as compared to the intervention group. In fact, small sample size and high heterogeneity may have had a significant effect on this finding. The difference in differences method was used and the mean changes in total motility in the placebo and the intervention group were compared. Finally, based on our results, no significant difference was found between the intervention and placebo groups. It seems that our interventions did not have significant effect on this parameter as compared with the placebo group.

Consistent with our results, Keskes-Ammar et al. (18) showed that the serum concentration of vitamin E in selenium (225 µg/day for three months) and vitamin E (400 mg/day for three months) treated group, significantly increased and malondialdehyde (MDA) significantly decreased. But no significant effects on serum MDA level and serum vitamin E concentration was detected in vitamin B treated group. However, no significant difference in sperm concentration was reported between the two groups. According to Keskes-Ammar et al. (18), dietary content of selenium (64.3 ± 17.7 µg/day) was lower than the amount reported in other studies (80-110 µg/day). Thus, sample size and short duration of follow-up may have a significant role as compared with other similar studies.

In another study, Scott et al. (19) indicated that sperm concentration in the selenium-treated group increased by 22%, in contrast with little or no increase in the group which received selenium + vitamin E + vitamin C + vitamin A and in the placebo group. They also reported that variations in response, small sample size and low concentration of administered selenium, prevented significant difference in sperm concentration among mentioned groups as compared with similar studies.

In another consistent study, Greco et al. (20) investigated the efficacy of two-month daily intake of 1g vitamin E and 1 g vitamin C on sperm parameters in infertile men. At the end of the study, no significant improvement in sperm motility was found. Small sample size, short duration of intervention and high-dose of antioxidants (which has a reverse effect) may have affected their results.

On the other hand, in consistent with our results, in a case-control study, Eroglu et al. (21) indicated lower serum and seminal concentration of selenium and semen total antioxidant capacity (TAC) in patients with oligozoospermia as compared with men with normozoospermia. In fact, selenium has a significant role in the production of mature spermatozoa from immature spermatids. Also, it contributes to the formation of glutathione peroxidase as an important enzyme in the mid-piece of human spermatozoa and protecting spermatozoa against oxidative stress, finally improving sperm quality.

Another study showed that vitamin E combined with clomiphene citrate, as antioxidant and anti-estrogen therapy, were more efficient in improving sperm concentration in idiopathic oligoasthenozoospermia compared with each one individually (22). It was concluded that vitamin E as an antioxidant acts more efficiently in combination of an antiestrogenic hormone such as clomiphene citrate, in improvement of sperm concentration in comparison with its individual administration. 

Our study showed no significant improvement in total sperm motility and progressive sperm motility between the two groups.

Consistent with our study, Hassani-Bafrani et al. (23) investigated the effect of vitamins E and B and their combination on sperm motility in a rat varicocele model. They find no significant improvement in sperm motility in varicocelized rats treated with vitamin E. Meanwhile, a significant improvement in vitamin B and vit B+E varicocelized treated group was detected. It is likely that these improvements were due to B complex specially B12 rather than vitamin E alone. In fact, vit B12 plays an important role in the regeneration of one carbon cycle and methionine synthesis and thermoregulation of scrotal and finally improvement of sperm parameters.

In consistence with our results, Moslemi and Tavanbakhsh (24) indicated a significant improvement in sperm motility of asthenoteratozoospermia patients who received daily supplements of selenium and vitamin E for at least 100 days (compared with the baseline). In fact, selenium and glutathione contribute to production of phospholipid hyperoxide GSH-Px, a structural protein that contains more than 50% of mitochondrial capsule of spermatozoa mid-piece, the deficiency of which leads to instability of spermatozoa mid-piece and finally, asthenozoospermia. It seems that the difference in results may partly be due to the larger sample size and longer period of this study in comparison with our study

Another study showed that total sperm motility increased by 40 and 34% in selenium-treated and B complex-treated groups, respectively. Also, it was reduced by 15% in the placebo group, however, differences among these three groups were not significant. When seleniumtreated and B complex-treated groups were combined and compared with the placebo group, a significant improvement in the selenium-treated group was found in comparison with the control group (19). Significant improvements in sperm motility in a larger sample size of men taking selenium supplementation, were indicated.

In agreement with our study, Hawkes et al. (25) evaluated the effect of 48-weekdaily intake of 300 µg of selenium on sperm parameters in healthy volunteer men. Although selenium concentration increased to 61% in blood plasma and 49% in seminal plasma, selenium supplementation had no significant improving effect on serum androgen concentration or sperm motility. It was concluded that testes are as well-protected from selenium excess as well as selenium deficiency. Hence, consumption of a high level of selenium does not change selenium content of sperm and sperm parameters. 

Consistent with our results, a systematic review and meta-analysis reported no significant difference in the sperm motility in the folate supplemented group compared with the control group. Also, there was no significant difference in sperm motility in folate plus Zn group in comparison with the control group. Zinc deficiency reduces the absorption and metabolism of dietary folate because it works as a cofactor for the folate-metabolizing enzymes dihydrofolate reductase and y-glutamyl hydrolase. Therefore, a combination of folic acid and zinc work more efficiently than when they are taken alone (26).

In our study, no significant difference was found in sperm normal morph between the intervention and placebo groups.

Compatible with our results, Raigani et al. (27) showed that although seminal concentration of folic acid in folic acid and folic acid plus zinc sulfate groups was significantly improved as compared with B complex treated group, there was no significant difference in sperm normal morph among groups. One could say that the results of the above study are probably due to lack of control over the nutritional status. In other words, inappropriate diets, smoking, alcohol consumption and exposure to environmental contaminants have certainly had significant roles in the above study.

In consistence with our study, Mohammadi et al. (28) investigated the effect of Condensyl (B vitamins, N-acetyle cysteine, zinc, small amount of vitamin E and quercetine) as a complex of antioxidants to improve sperm parameters, two months after surgical varicocele induction in rats. A significant improvement in testis characteristics and considerable improvements in sperm morphology, were indicated. Folic acid in combination with B2, B3, B6 and B12, supports the one carbon cycle homocysteine re-methylation and increase the efficiency of one carbon metabolic cycle, and finally, reduction of spermatozoa damage in infertile men.

However, another study showed a significant improvement in sperm normal morph after selenium and vitamin E consumption in patients with asthenoteratozoospermia (24). In fact, the large sample size in the mentioned study and longer period of intervention in comparison to our study may have a considerable role in this significant statistical result.

The beneficial effect of antioxidant therapy to treat oxidative-stress-induced male infertility has been indicated in some studies. On the other hand, it can be claimed that reductive stress can be dangerous to cells as oxidative stress. It would be accrued due to ignoring the assessment of the redox status in infertile patients and over use of antioxidants. In these cases, subsequent to antioxidant therapy, endogenous oxidants which are necessary for sperm maturation reduced considerably (10).

In an evidence-based review by Ahmadi et al. (29), it was shown that administration of supplementations such as vitamin E and C, selenium and L-carnitine may ameliorate sperm concentration, motility, morphology and sometimes DNA integrity, but further clinical researches are recommended in order to determine appropriate antioxidant component and efficient antioxidant dose.

Similar to our study, Ardestani Zadeh et al. (13) investigated the effect of daily intake vitamin E) 400 mg, oral, daily) and folic acid (5 mg, oral, daily) and selenium (200 µg, oral, daily) on sperm parameters in 64 infertile men who underwent varicocelectomy and finally, contrary to our results, a significant increase in sperm concentration and motility was reported in the intervention group. This discrepancy with our results may be due to the type of patients studied, duration of intervention and the difference in the semen analysis device. They used anoptical microscope for semen analysis and infertile men were studied and treated with antioxidants for six months.

In another similar study, Moslemi and Tavanbakhsh (24) investigated the effect of daily intake of selenium (200 µg, daily) and vitamin E (400 mg, daily) given for at least 100 days, on sperm parameters in people with idiopathic infertility. Unlike our findings, they found a significant increase in sperm motility and normal morphology compared to before the intervention. Contrary to our study, their study had a higher sample size (690 people), and a longer period of antioxidant therapy but did not have a control group and treatment with placebo. Moreover, they used an optical microscope.

Using an effective component of antioxidants based on their special mechanism, blinded participants and using SQAV as an analytical medical device which performs a complete quantitative evaluation of semen quality and semen parameters in less than 2 minutes. SQAV is also a high-performance analyzer that incorporates technology in electro-optics, computer algorithms and video microscopy and provides a quick, precise and accurate automated semen analysis.

Short duration of intervention, small sample size, lack of access to combined antioxidant supplementation of selenium, vitamin E and folic acid at the mentioned dose and lack of access to similar shape and structure placebo.

## Conclusion

Our findings indicated that consumption of selenium, vitamin E and folic acid in infertile men with asthenozoospermia was not effective. However, further prospective randomized controlled trials with a larger sample size, that evaluate semen oxidative status before starting antioxidant therapy, are recommended to confirm the effectiveness of antioxidant therapy on sperm parameters in males with idiopathic infertility.
